# Neurosyphilis with dementia and bilateral hippocampal atrophy on brain magnetic resonance imaging

**DOI:** 10.1186/1471-2377-12-96

**Published:** 2012-09-20

**Authors:** Shima Mehrabian, Margarita Raycheva, Martina Traykova, Tonya Stankova, Latchezar Penev, Olga Grigorova, Latchezar Traykov

**Affiliations:** 1 University Hospital “Alexandrovska”, Department of Neurology, 1, Georgi Sofiiski str, 1431 Sofia, Bulgaria; 2 Department of Radiology, University Hospital “Alexandrovska”, Sofia, Bulgaria

**Keywords:** Neurosyphilis, Dementia, Hippocampal atrophy, Alzheimer’s disease, Magnetic resonance imaging (MRI)

## Abstract

**Background:**

This article reports a rare case of active neurosyphilis in a man with mild to moderate dementia and marked hippocampal atrophy, mimicking early onset Alzheimer’s disease. Few cases have so far described bilateral hippocampal atrophy mimicking Alzheimer’s disease in neurosyphilis.

**Case presentation:**

The patient presented here is a 33 year old Bulgarian male, whose clinical features include progressive cognitive decline and behavioral changes over the last 18 months. Neuropsychological examination revealed mild to moderate dementia (Mini Mental State Examination score was 16/30) with impaired memory and attention, and executive dysfunction. Pyramidal, and extrapyramidal signs, as well as dysarthria and impairment in coordination, were documented. Brain magnetic resonance imaging showed cortical atrophy with noticeable bilateral hippocampal atrophy. The diagnosis of active neurosyphilis was based on positive results of the Venereal Disease Research Laboratory test/*Treponema pallidum* hemagglutination reactions in blood and cerebrospinal fluid samples. In addition, cerebrospinal fluid analysis showed pleocytosis and elevated protein levels. High-dose intravenous penicillin therapy was administered. At 6 month follow up, improvements were noted clinically, on neuropsychological examinations, and in cerebrospinal fluid samples.

**Conclusion:**

This case underlines the importance of early diagnosis of neurosyphilis. The results suggest that neurosyphilis should be considered when magnetic resonance imaging results indicate mesiotemporal abnormalities and hippocampal atrophy. Neurosyphilis is a treatable condition which requires early aggressive antibiotic therapy.

## Background

Neurosyphilis results from infection of the brain, meninges or spinal cord. The infection is caused by the bacteria *Treponema pallidum* and develops in about 25%–40% of persons who are not treated for syphilis. After the appearance of acquired immunodeficiency syndrome (AIDS) in 1981, the occurrence of neurosyphilis in human immunodeficiency virus (HIV) infection may explain the increased number of new cases in developed countries. Neurosyphilis is treatable with antibiotics, however, early diagnosis and treatment is critical. Making the diagnosis is often difficult given the wide variety of central nervous system (CNS) manifestations of neurosyphilis, both clinically and on neuroimaging. The clinical presentations of neurosyphilis are extremely diverse and for practical purposes can be divided into early and late neurosyphilis. The clinical picture of meningovascular syphilis, the most common form of neurosyphilis, may be associated with focal neurological signs of cerebral arteritis [1,2].

Cognitive decline is one of the late syphilis manifestations. However, mild cognitive impairment has been observed in early stages of neurosyphilis [3].

Neuroimaging findings of meningovascular syphilis include cortical and subcortical infarcts, cortical atrophy, hydrocephalus, leptomeningeal enhancement associated with a clinical meningitis, and arteritis. There are two forms of arteritis: Heubner arteritis, which is the more common form affecting the medium and large arteries, and Nissl-Alzheimer arteritis, which affects the small arteries and arterioles. Other manifestations of meningovascular syphilis include leptomeningeal and cerebral gummas [1,4-6].

Few case reports have presented mesiotemporal abnormalities in neurosyphilis [7].

This article reports a rare case of active neurosyphilis in a man with mild to moderate dementia and marked hippocampal atrophy, mimicking early onset Alzheimer’s disease (EOAD).

## Case presentation

We present a case of a 33 year old Bulgarian male who attended a Neurological Clinic at University Hospital Alexandrovska. He attended the clinic because of progressive attention and memory impairments as well as apathy, anxiety and irritability reported by family members. Eighteen months earlier his brother noticed that he was confused, forgetful and unable to manage his professional activities as a construction worker. He became apathetic, irritable and more verbally aggressive. Our patient has a low level of education with poor written language and simple arithmetic abilities; however he was a good worker who was able to carry out everyday activities.

Neuropsychological assessments were performed using a general cognitive functioning scale (Mini Mental State Examination, MMSE), and tests of attention, memory, language and executive functions (see Table 1). Our patient was not fully orientated (he did not know the year, season, month, day and date), however, he was relatively orientated to place. Assessment of memory revealed a severe verbal learning impairment with an extremely low ability to retain new information. He also demonstrated difficulty with remembering autobiographical and personal information. His verbal communication was relatively spare; however he did have a mild anomia and poor categorical fluency. A severe dysexecutive syndrome was also documented (with notably poor coding test and letter fluency). Bearing in mind the patient’s premorbid cognitive functioning, neuropsychological assessment revealed mild to moderate dementia (MMSE = 16/30).

**Table 1:** Neuropsychological follow-up

**Neuropsychological assessment**	**I examination**	**II examination – after 6 months**
**MMSE**	16	19
**Verbal memory**		
**(10 words)**		
Immediate recall	4	4
Delayed recall	1	2
Recognition	0	2
**Language**		
VF (animal)	10	13
BNT	10	10
**Attention/executive function**		
Digit symbol (coding)	8 (2 mistakes)	10 (1 mistakes)
VF (M)	1	3

*MMSE* - Mini Mental State Examination; *BNT* – Boston Naming Test; *VF* (animal) – Verbal Fluency, number of animals produced for 1 min; *VF (M)* – Verbal Fluency, number of words beginning with letter “M” for 1 min.

The patient had no history of skin lesions or symptoms of Argyll-Robertson; his pupillary reflexes were preserved, and the pupils constricted in response to light and accommodation. Pyramidal and extrapyramidal signs, dysarthria, and impairment in coordination were documented.

**Figure 1:**
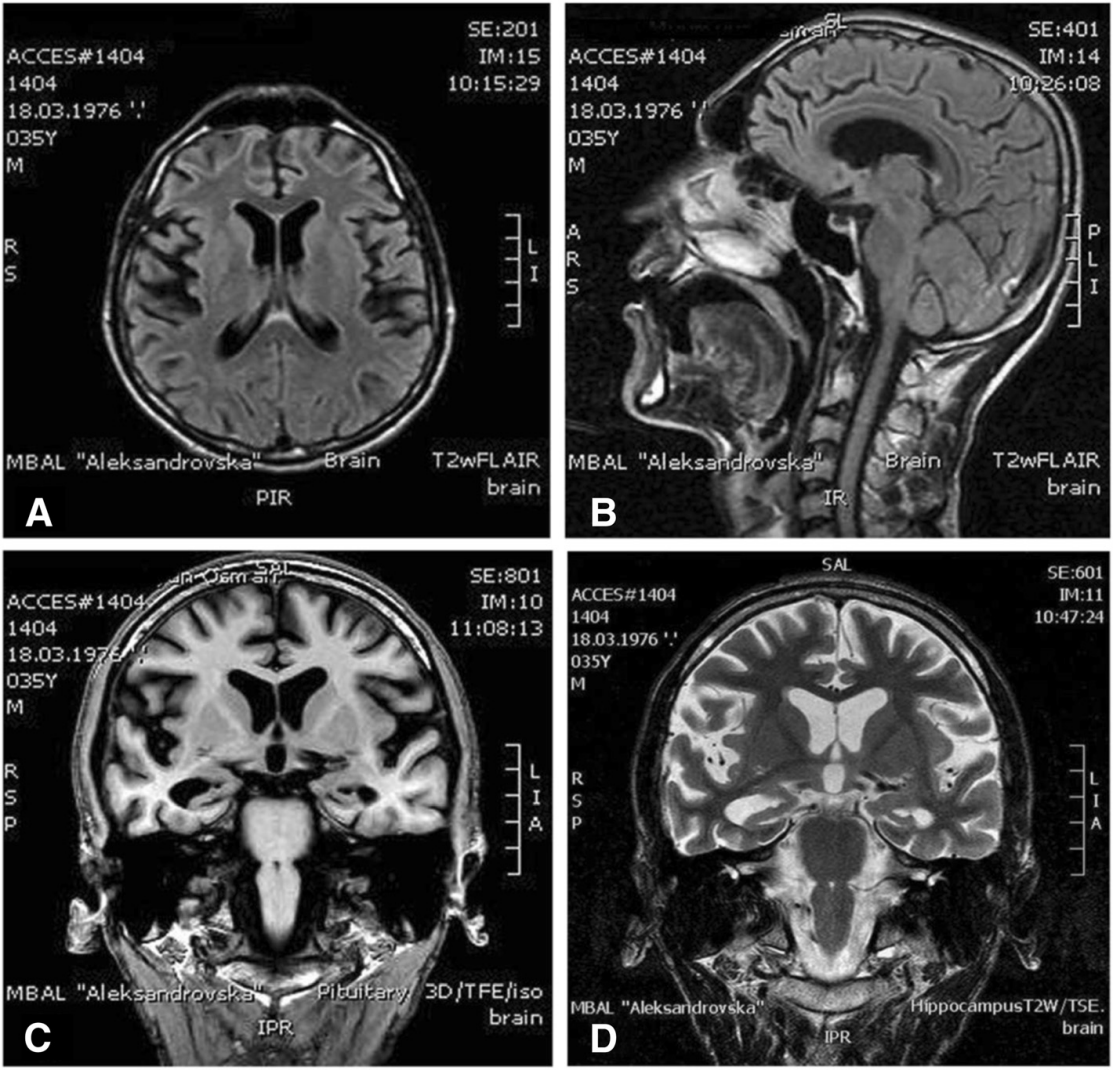
**Magnetic Resonance Imaging (MRI) of the brain. A**/ Axial fluid attenuated inversion recovery (FLAIR). **B**/ Sagittal fluid attenuated inversion recovery (FLAIR). **C**/ Coronal 3D -T1-TFE. **D**/Coronal T2WI – TSE. All images are showing marked diffuse loss of brain parenchyma including mesiotemporal atrophy. Note that there are no areas of increased signal intensity in the FLAIR and T2W images.

Laboratory workups, including a complete and differential blood count, serum electrolytes and glucose, liver and renal function tests, thyroid function tests, serum B12 and folate levels, were normal. In addition, cerebrospinal fluid (CSF) analysis showed pleocytosis, elevated protein levels, and positive oligoclonal bands. Cerebrospinal fluid was clear with 1 × 10^6^/l erythrocytes, 39 × 10^6^/l leucocytes (82% lymphocytes, 16% monocytes) and a protein level of 0.88g/l. The diagnosis of active neurosyphilis was based on positive results of the Venereal Disease Research Laboratory test/*Treponema pallidum* hemagglutination assay (VDRL/TPHA) reactions in blood and CSF samples (serum-VDRL 1:128, serum-TPHA 1:2560, CSF-VDRL 1:64, CSF-TPHA 1:640). The serum and CSF test for HIV was negative. Magnetic resonance imaging (MRI) of the brain demonstrated moderate cortical and marked hippocampal atrophy (Figure 1).

No adverse reactions were observed upon receiving a course of intravenous Penicillin G 5x5000000 IU /daily for 20 days.

At the 6 month follow up examination, clinical signs and neuropsychological findings showed slight improvement in general cognitive functioning. Six months after treatment the patient’s MMSE score was 19/30 (see Table 1). Improvement was also noted on the activities of daily living and behavioural disturbances assessments. The patient’s CSF protein level was 0.55 g/L, with 1 × 10^6^/l leucocytes. The VDRL test of CSF indicated positive results.

## Discussion

We have presented a case of active neurosyphilis with dementia, behavior changes and rare bilateral hippocampal atrophy mimicking EOAD.

A few reports have described the mesiotemporal involvement in neurosyphilis. Several reports have documented unilateral or bilateral asymmetrical mesiotemporal T2 hyperintense lesions on MRI images of the brain in neurosyphilis. This is especially important considering that the clinical presentation of neurosyphilis may also seemingly mimic that of herpes simplex encephalitis or paraneoplastic limbic encephalitis [5,7-10].

The cause of the mesial temporal T2-weighted hyperintensity is not clear. To our knowledge, there is no pathological study on mesial temporal hyperintensity on T2 weighted images in patients with neurosyphilis. It is suggested that the signal changes represent a combination of edema and gliosis. There are most likely multiple causes for the edema component itself. In meningitis, there are changes in the meningeal and cerebral capillaries, which may increase permeability of the blood–brain barrier, leading to a vasogenic edema component. There may also be some cytotoxic edema and an interstitial edema component, inflammation, meningovasculitis, or microglial hypertrophy [5,7].

There is no reliable marker to predict the outcome in patients with neurosyphilis following treatment. The duration the illness takes to become symptomatic and the delay in therapeutic intervention are important factors for preventing further progression of the disease. Cases with reversible high signal T2 lesions in the mesial temporal region have been reported [11-13]. The presence of edema is further suggested by the imaging improvement which was found consequent to antibiotic therapy. The residual T2 hyperintense lesion may have a vasculitic origin. The presence of gliosis may be secondary to infection-induced small-vessel ischemic changes.

In our patient, the cognitive and behavioral symptoms, neuropsychological profile, MRI data, and the early age at onset, were compatible with a diagnosis of probable EOAD. Moreover, focal neurological abnormalities have been reported in a rare case of EOAD [14]. However, the serological tests were performed because of the young age and focal neurological abnormalities.

Neurosyphilis is associated with cognitive decline and progressive dementia. A small number of cases have been described mimicking Alzheimer’s disease radiologically. In one of the four cases reported by Zifko et al., MRI of the brain showed atrophy and gliosis over bilateral hippocampi [15]. Another patient reported by Van Eijsden et al. [16] presented with cognitive and behavioral symptoms. On the MRI brain scan there was medial temporal lobe atrophy which was equivalent to the highest degree of atrophy on a visual rating scale, mimicking EOAD. Our patient showed similar neuropsychological and neuroimaging features with the case described above. Both cases had AD like neuropsychological profiles and a high degree of medial temporal atrophy. Additionally, our patient had focal neurological signs (pyramidal and extrapyramidal signs). CSF abnormalities in both cases showed pleocytosis and elevated protein levels. The follow-up examination showed improvement in neuropsychological results after treatment in both cases.

It is not clear why atrophy occurs in the medial temporal regions. One explanation is that initial infection causes inflammatory T2 hyperintensity which, with no treatment, may disappear at a later stage when the tissues undergo irreversible atrophy [16]. Another explanation may be the emergence of secondary atrophy due to dysfunction in the diffuse cerebral cortex; the medial temporal regions have connections with the cerebral association areas, including the frontal, temporal, and parietal lobes.

This is the first case report of a Bulgarian patient with neurosyphilis, showing progressive cognitive and behavioral changes with bilateral cortical and hippocampal atrophy mimicking EOAD.

## Conclusion

The temporal lobe imaging abnormalities described here suggest the necessity of including neurosyphilis in differential diagnosis of medial temporal lobe T2 hyperintensities (herpes simplex encephalitis or paraneoplastic limbic encephalitis) and mesiotemporal atrophy (Alzheimer’s disease). Reversible MRI lesions in patients with neurosyphilis after treatment correlates with clinical improvement. This should alert physicians to the fact that early diagnosis and aggressive treatment are worthwhile.

## Consent

Written informed consent was obtained from the patient for publication of this case report and any accompanying images. A copy of the written consent is available for review by the Editor-in-Chief of this journal.

## Abbreviations

AIDS: Acquired immunodeficiency syndrome; HIV: Human immunodeficiency virus; CNS: Central nervous system; EOAD: Early onset Alzheimer’s disease; MMSE: Mini Mental State Examination; CSF: Cerebrospinal Fluid; VDRL-TPHA: Venereal Disease Research Laboratory test - Treponema Pallidum. Hemagglutination Assay; MRI: Magnetic Resonance Imaging.

## Competing interests

The authors declare that they have no competing interests.

## Authors’ contributions

All authors participated in the care of the described patient. SM and MR were major contributors in writing the manuscript. MT, TS, LP and OG collected the data and helped to draft the manuscript. LT critically revised the content of this manuscript. All authors have read and approved the final version of the manuscript.

## Pre-publication history

The pre-publication history for this paper can be accessed here:

http://www.biomedcentral.com/1471-2377/12/96/prepub
